# Combining Default Choices and an Encounter Decision Aid to Improve Tobacco Cessation in Primary Care Patients: A Pragmatic, Cluster-Randomized Trial

**DOI:** 10.1007/s11606-024-09088-9

**Published:** 2024-10-09

**Authors:** Kevin Selby, Inès Habfast-Robertson, Marie-Anne Durand, Christina Hempel-Bruder, Anne Boesch, Joachim Marti, Yasser Kazaal, Mohamed Faouzi, Hubert Maisonneuve, Ivan Berlin

**Affiliations:** 1 Department of Ambulatory Care, University Center for Primary Care and Public Health (Unisanté), University of Lausanne, Rue de Bugnon 44, 1011 Lausanne, Switzerland; 2 The Dartmouth Institute for Health Policy & Clinical Practice, Dartmouth College, Lebanon, NH USA; 3 Department of Addiction Medicine, CHUV, Lausanne, Switzerland; 4 Faculty of Medicine, University Institute for Primary Care, University of Geneva, Geneva, Switzerland; 5 University College of General Medicine, University Claude Bernard Lyon 1, Lyon, France; 6 Département de pharmacologie médicale, Hospital Pitié-Salpêtrière-Sorbonne Université, Paris, France; 7 Department of Epidemiology and Health Systems, University Center for Primary Care and Public Health (Unisanté), University of Lausanne, Lausanne, Switzerland; 8 Division of Biostatistics, University Center for Primary Care and Public Health (Unisanté), University of Lausanne, Lausanne, Switzerland

**Keywords:** smoking cessation, primary care, decision aid, default choice

## Abstract

**Background:**

Primary care providers (PCPs) prescribe less often treatments for smoking cessation than for other major risk factors. We assessed the effect of training PCPs to offer smoking cessation treatments to current smokers as the default choice using an encounter decision aid (DA) on smoking cessation.

**Methods:**

Pragmatic, cluster-randomized controlled trial with PCPs in private practice in Switzerland and France. The intervention was a half-day course teaching PCPs the default choice approach using a DA. Control PCPs received a 1-h refresher training on smoking cessation aids. PCPs recruited daily smokers seen for routine care. The primary outcome was self-reported, 7-day, point prevalence smoking abstinence at 6 months. Secondary outcomes were quit attempts and use of smoking cessation aids at 3 weeks, 3 months, and 6 months, and a patient-reported measure of shared decision-making (CollaboRATE scale 1–10, higher scores = more involvement).

**Results:**

Forty-two PCPs completed the training (76% Swiss) and recruited 287 current smokers (105 intervention group, 182 control group), with 51% women, mean age 48 (SD, 2.6), 77% who smoked <20 cigarettes/day, and 221 who responded at 6 months follow-up (77%). The intervention did not affect self-reported smoking abstinence rate at 6 months (9.5% intervention and 10.4% control groups, respectively; OR 0.88 (95%CI 0.37–2.10). It did however increase the number of quit attempts at 3 weeks (OR 2.09, 95%CI 1.04–4.20) and the use of smoking cessation aids at the 3-week and 3-month follow-ups (OR 2.57, 95%CI 1.21–5.45 and OR 2.00, 95%CI 1.11–3.60, respectively). The mean CollaboRATE score was 8.05/10 in the intervention group and 7.28/10 in the control group (*p*=0.02), reflecting more patient involvement in decision-making.

**Conclusion:**

Training PCPs to use a decision aid did not improve smoking abstinence rate, despite short-term increases in quit attempts and use of smoking cessation aids. It improved patient involvement in decision-making.

**Trial Registration:**

ClinicalTrials.gov identifier: NCT04868474.

**Supplementary Information:**

The online version contains supplementary material available at 10.1007/s11606-024-09088-9.

## INTRODUCTION

Tobacco smoking led to 7.7 million deaths worldwide in 2019 and remains the leading cause of preventable mortality in Switzerland.^1^ Quitting smoking at age 35 to 44 or 45 to 54 years provides a gain in life expectancy of 9 and 6 years, compared to continuing to smoke.^2^ Multiple interventions can be delivered in primary care to promote smoking cessation, such as providing advice,^3^ prescribing pharmacological therapy,^4^ and possibly encouraging use of electronic cigarettes.^5^

However, primary care providers (PCPs) less often provide medications or counselling for tobacco use than other major risk factors (e.g., hypertension, diabetes).^6^ PCPs often inquire about tobacco consumption and provide brief advice, but rarely prescribe treatments or provide help to quit over repeated visits.^7^ This may in part be due to several guidelines recommending PCPs to only offer quit support to patients who are motivated to quit and “opt-in.”^8^ However, motivation to quit is not stable over time^9^ and is a poor predictor of willingness to discuss quitting.^10,11^ An alternative approach is to discuss treatments for smoking cessation as the default choice for all current smokers, with possibility to “opt-out,” similar to how treatments are presented for high blood pressure or diabetes.^12,13^ Defaults can have a strong impact on choices and treatment use.^14^

Previous research has suggested that presenting smoking cessation counselling^15^, treatment^16^, and referral^17^ as the default choice convincingly increases uptake and may improve tobacco cessation. However, interventions to date have been limited to hospitalized patients and pregnant women; implementation of such an approach in primary care could have an even bigger impact given the frequency of contacts with current smokers. Motivation to quit is not a predictor of patients’ openness to discuss treatments or quit success after controlling for nicotine dependence.^18^,^19^ A promising alternative would be to discuss smoking cessation with all current smokers, reserving brief interventions for those who refuse a discussion and motivational interviewing for those who express ambivalence.^13^

A potential downside of this approach is a resurgence of medical paternalism and a failure to involve patients in a sensitive decision.^20^ Explicitly involving patients with a decision aid (DA) could offset that effect and improve patient involvement. DAs are “designed to help patients make specific and deliberate choices from among healthcare options; they are intended to supplement (rather than replace) clinicians' counseling about options.”^21^ They improve patient knowledge, participation in decision-making, and congruency between informed values and care choices compared to usual care, and are typically used either to prepare for a clinician consultation or during the consultation (encounter DAs).^21^ DAs can also increase provider knowledge and focus discussions;^22^ PCPs often cite lack of time and insufficient experience prescribing smoking cessation medications as barriers to treating tobacco use.^23^

Few studies have evaluated DAs for smoking cessation, with a trend towards an increase in quit attempts.^24^ Two studies evaluated encounter DAs, including one randomized trial of 130 patients scheduled for elective surgery.^25^ The DA improved decision quality but not behavior; however that DA focused on the decision whether to quit and not treatments.^25^ We created an encounter DA comparing smoking cessation treatments^22,26^ and observed anecdotally that it provided an acceptable means of presenting treatment options to smokers regardless of their level of motivation.

Given the potential of default choices to increase the number of discussions about smoking cessation and of the DA to augment patient involvement, we thought their use together could trigger more quit attempts and facilitate the prescription of smoking cessation aids, resulting in higher rates of tobacco abstinence at 6-months follow-up. The aim of this trial was thus to assess the effect of training PCPs to offer smoking cessation treatments as the default choice using an encounter DA.

## METHODS

### Design and Setting

We conducted a cluster-randomized, controlled, superiority trial with 1:1 allocation of PCPs who then recruited patients seen in consultation. The trial was conducted in French-speaking Switzerland and the Rhone-Alps region of France between June 2021 and October 2023. The PCPs in Switzerland all worked in small, physician-owned practices (2 to 10 physicians) with medical assistants, but no nurses. Switzerland has universal, mandatory private health insurance with variable deductibles. The PCPs in France worked in multidisciplinary group practices that integrate public health nurses. France has universal public health insurance. The trial was registered prior to inclusion of the first participant (NCT04868474) and the protocol published.^27^ The trial was approved by the Vaud Ethics Committee (2020–02.898) and received an exemption from the National College of Teachers in General Practice (CNGE) in France (Decision 121222435). We followed CONSORT reporting guidelines.^28^

### Participants

We recruited PCPs in private practices with >80 patients per month. PCP-level exclusion criteria were recent training in smoking cessation and plans to retire or relocate within <12 months. After the training program, PCPs were instructed to recruit among patients who were current smokers presenting for routine care. Patient-level inclusion criteria were age ≥18 years old, used tobacco daily (cigarettes, cigars, smokeless tobacco), and considered the recruiting PCP as their primary care doctor. Patient-level exclusion criteria were an acute medical condition that precluded even a brief discussion of smoking cessation, inability to provide informed consent (including due to language barriers as materials were in French), recent enrolment in a smoking cessation trial, and current daily use of a pharmacologic smoking cessation aid including electronic cigarettes. PCPs were encouraged to discuss smoking cessation with their patient during the baseline visit, if time permitted, using either the default choice with DA (intervention), or their usual approach (control). Follow-up visits to discuss smoking cessation were at the discretion of the PCPs, while all follow-up information was collected by telephone by the research team, without attempting to influence patients’ decision to quit smoking. PCPs received compensation for participating in the training, completing follow-up questionnaires, and per patient enrolled. Patients were not compensated.

### Intervention and Control

The general approach has been described elsewhere.^13,27^ Briefly, the intervention consisted of a half-day training with two parts. First, a 1.5-h presentation focusing on the benefits and use of pharmacological and non-pharmacological smoking/tobacco cessation aids, as well as the concept of presenting tobacco cessation aids as the default choice with an encounter decision aid. The encounter decision aid was designed and tested by the study authors and made available in both paper and electronic forms.^22^ And second, a demonstration of the default choice technique with a video, followed by role plays with common scenarios from primary care to use default choices and the electronic decision aid.

The control group received the first 45 to 60 min of the intervention, primarily how to use pharmacological and non-pharmacological tobacco cessation aids, but without use of the DA. Both groups were trained to identify current smokers eligible for the trial and complete the informed consent.

### Outcomes

The primary outcome was self-reported 7-day, point prevalence smoking abstinence at 6 months recorded during a telephone interview. Patients not responding to multiple telephone calls or email prompts to complete the questionnaire online were contacted by text messaging requesting if they had smoked tobacco in the previous 7 days. Secondary outcomes were the point-prevalence smoking abstinence, quit attempts since the last follow-up, and use of smoking cessation aids since the last follow-up, measured at 3 weeks, 3 months, and 6 months, and the patient-reported CollaboRATE scale (scale 1–10, higher scores = more involvement).^29^ The CollaboRATE scale contains three questions developed in English and validated in French: how much the provider helped the patient understand, listened to what matters most, and included what matters most to them in choosing what to do next.^29^ Important changes to the protocol were expanding recruitment into France, stopping offering carbon monoxide testing for those having quit at 6 months follow-up (very few patients willing to attend in person), and ending the trial prematurely due to inadequate recruitment. Despite extending the recruitment period, most PCPs recruited fewer patients than planned and we did not have sufficient resources to recruit additional PCPs.

### Sample Size

We hypothesized that in the control group, 10% of current smokers would be recommended a smoking cessation aid and 4% would successfully quit.^30^ We thought that 10.5% of patients would successfully quit with the default choice approach (odds ratio of 2.8). If each arm had 20 clusters with 20 patients each, we would achieve 81.4% power to detect this difference with an intracluster correlation of 0.03. Assuming 15% drop out in both groups, we aimed to include 46 PCPs recruiting 23 patients each, so 1058 patients in total (that is 529 by group).

### Randomization and Blinding

Randomization was done by the study statistician (MF) in blocks of two to four PCPs. PCPs from the same practice were randomized together. PCPs were blinded, as all materials mentioned just two versions of a training about smoking cessation, and not the nature of the intervention. Patients were likewise told that the study compared two training programs of their physicians. Outcome assessors were not blinded to study arm when performing follow-ups with PCPs and patients; however, care was taken not to reinforce teachings related to the intervention (i.e., use of the decision aid or default choices). The study statistician was blinded to group assignment.

### Statistical Methods

PCP and patient characteristics were described using proportion or means with standard deviation as appropriate. The primary outcome was analyzed using an intention to treat approach with logistic regression controlling for clustering by the recruiting PCP, with persons lost to follow-up considered as still smoking. All secondary outcomes were analyzed with logistic regression controlling for clustering by the recruiting PCP, calculated with complete data only, assuming missing data were missing completely at random. Data were analyzed using Stata 18 (StataCorp. 2023, Stata Statistical Software: Release 18, College Station, TX).

## RESULTS

### Enrollment and Characteristics of Participants

**Figure 1:**
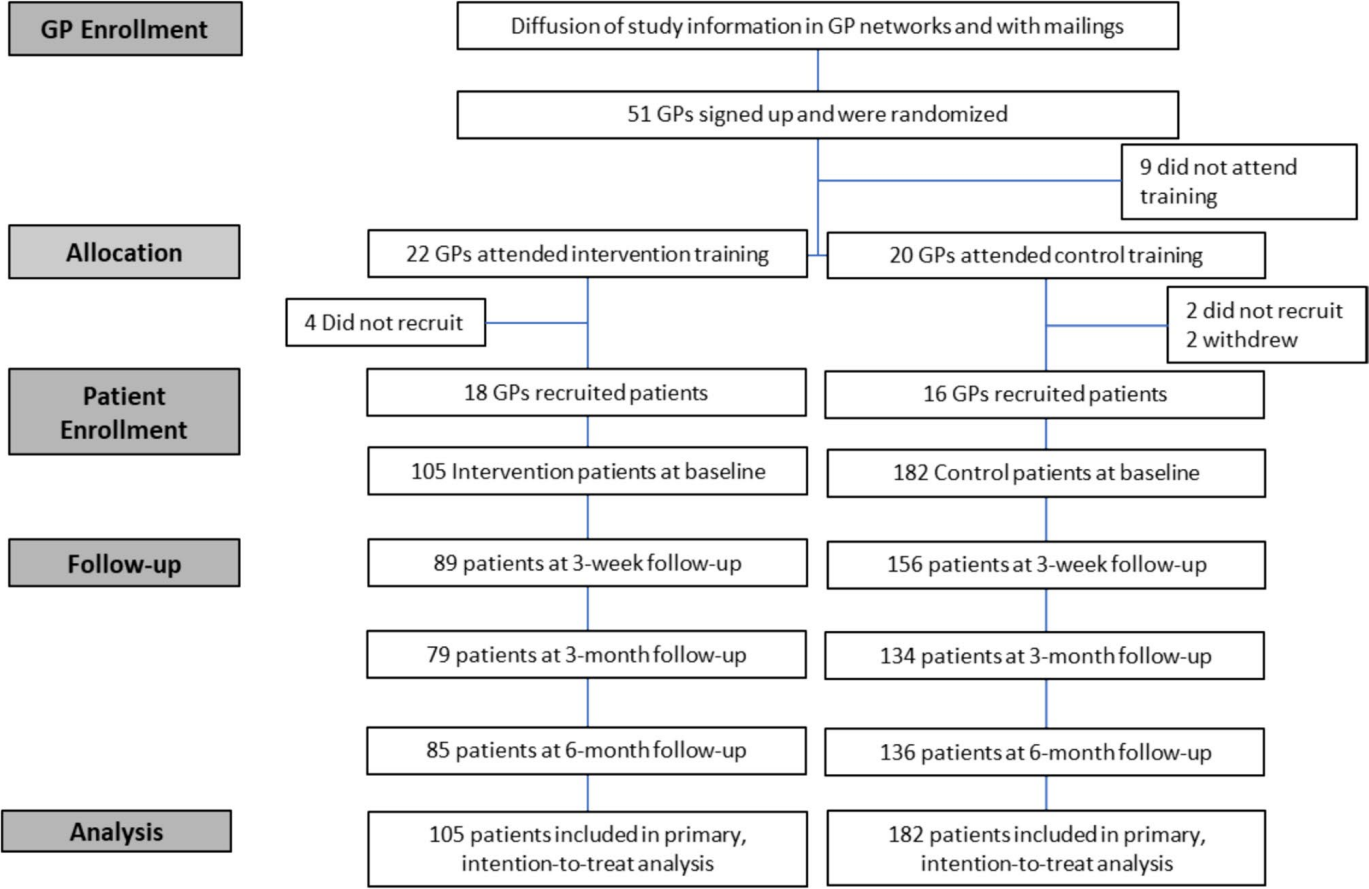
CONSORT diagram.

Fifty-one PCPs were invited to participate and randomized to a training date. Nine withdrew prior to consent (4 intervention and 5 control group), such that 42 PCPs (82%) completed the training (Fig. 1 and Table 1). There were 26 women (62%), with 19 aged between 40 and 49 years (45%), and 32 had their practice in Switzerland (76%). At baseline, most physicians used motivational interviewing. Eight PCPs (4 intervention and 4 control) did not recruit patients. The 34 remaining PCPs recruited between 1 and 25 patients (Supplemental Figure 1 shows distribution of patients per PCP).

**Table 1:** Baseline Characteristics of Participating Primary Care Providers (PCPs, *n*=42) and Patients (*n*=287)

**PCP characteristics**	**Intervention** (*n*=22)	**Control** (*n*=20)	**Total** (*n*=42)
Age			
30–39 years	7 (32%)	7 (35%)	14 (33%)
40–49 years	9 (41%)	10 (50%)	19 (45%)
50–64 years	6 (27%)	3 (15%)	9 (22%)
Gender			
Men	9 (41%)	7 (35%)	16 (38%)
Women	13 (59%)	13 (65%)	26 (62%)
Practice country			
Switzerland	17 (77%)	15 (75%)	32 (76%)
France	5 (23%)	5 (25%)	10 (24%)
Practice location			
Urban	17 (77%)	14 (70%)	31 (74%)
Rural	5 (23%)	6 (30%)	11 (26%)
**Patient** **characteristics**	**Intervention** (*n*=105)	**Control** (*n*=182)	**Total** (*n*=287)
Age (mean, SD)	47.2 (15)	49.1 (14)	48.4 (14)
18–29 years	14 (13%)	22 (12%)	36 (13%)
30–45 years	34 (33%)	46 (25%)	80 (28%)
46–65 years	43 (41%)	90 (49%)	133 (47%)
66+ years	13 (13%)	24 (13%)	37 (13%)
Gender			
Men	50 (49%)	90 (49%)	140 (49%)
Women	53 (51%)	92 (51%)	145 (51%)
Nationality			
Swiss	48 (46%)	112 (61%)	160 (56%)
French	24 (23%)	7 (4%)	31 (11%)
Other	33 (31%)	63 (35%)	96 (33%)
Health literacy*			
Low	15 (15%)	17 (10%)	32 (11%)
High	87 (85%)	162 (90%)	249 (89%)
Level of education			
Obligatory education or less	30 (29%)	33 (18%)	63 (22%)
Apprenticeship or high school†	45 (44%)	96 (53%)	141 (50%)
Post-secondary education	28 (27%)	52 (29%)	80 (28%)
Tobacco products used daily			
Cigarettes	97 (92%)	157 (86%)	254 (89%)
Cigars/cigarillos	2 (2%)	7 (4%)	9 (3%)
Heated tobacco products	2 (2%)	14 (8%)	16 (6%)
Other‡	13 (12%)	23 (13%)	36 (13%)
Used a nicotine product without tobacco in the last month	20 (19%)	46 (25%)	66 (23%)
Number of cigarettes or equivalent per day			
0–9	40 (38%)	65 (36%)	105 (37%)
10–19	38 (37%)	77 (42%)	115 (40%)
20 or more	26 (25%)	40 (22%)	66 (23%)
Time to first cigarette in the morning			
≤5 min	26 (25%)	68 (21%)	64 (22%)
6–30 min	41 (40%)	85 (47%)	126 (44%)
31–60 min	20 (19%)	24 (13%)	44 (15%)
>60 min	16 (16%)	35 (19%)	51 (18%)

^*^Low health literacy defined as answering, always or often difficulty when completing a medical form^34^

^†^Defined as having completed the matriculation examination without pursuing secondary education or having completed as least part of an apprenticeship

^‡^Other products included snus, Hookah, etc.

The PCPs recruited 287 current smokers (Fig. 1), of whom 51% were women. The patients’ mean age was 48 years (SD 14) and 77% smoked <1 pack of cigarettes per day (Table 1). The average Heaviness of Smoking Index, a self-reported measure including time to first cigarette and number of cigarettes per day, was 2.6/6 (SD 1.6). Further, 27% had made at least one quit attempt and 62% had the intention to try quitting in the next 3 months. Most variables appeared balanced between study arms, except nationality, with 23% of participants with French nationality in the intervention arm versus 4% in the control arm. In total, 221 (77%) responded at 6 months follow-up.

### Characteristics of the Baseline Consultations as Reported at 3-Week Follow-up

Most patients recalled discussing tobacco cessation in both groups (Table 2). The main motive of consultation was for routine follow-up (47%) and a check-up (30%). More participants in the intervention arm recalled having used a decision aid (50% vs 9%, *p*<0.001). More patients recalled being prescribed nicotine replacement therapy in the intervention group, but other treatments were similar. Varenicline was not available during most of the study (Pfizer pulled Champix in September 2021 and generic varenicline was not available).

**Table 2:** Patient-Reported Characteristics of Baseline Consultations, as Reported at 3-Week Follow-up

Outcome at 3-week follow-up	Intervention (*n*=89)	Control (*n*=156)	Total (*n*=245)	*p*-value
Patient recalls discussing tobacco cessation				*p*=0.803
Yes	76 (85%)	135 (87%)	211 (86%)	
No	13 (15%)	21 (13%)	35 (14%)	
Patient recalls of using decision aid				*p*<0.001
Yes	37 (51%)	11 (9%)	48 (24%)	
No	36 (49%)	113 (91%)	149 (76%)	
CollaboRATE score of baseline consultation				
Mean (SD)	8.05 (2.25)	7.28 (2.35)	7.55 (2.34)	*p*=0.02
Prescription of smoking cessation aids				*p*=0.037
Nicotine replacement therapy	29 (33%)	29 (19%)	58 (24%)	
Varenicline*	1 (1%)	0 (0%)	1 (0.3%)	
Bupropion	0 (0%)	3 (2%)	3 (1%)	
Electronic cigarette	11 (12%)	14 (9%)	25 (10%)	
Other treatment	4 (4%)	6 (4%)	10 (4%)	
No treatment or I don’t know	38 (43%)	93 (60%)	131 (53%)	

^*^Treatment removed from the market as of 2021 due to impurities found, therefore not commercially available as planned

### Primary and Secondary Outcomes

The intervention did not affect self-reported smoking abstinence rate at 6 months in the intention to treat analysis, with 9.5% abstinent in the intervention and 10.4% in the control groups, OR 0.88 (95%CI 0.37–2.10, Table 3). The complete-case analysis gave lower odds of abstinence with similar confidence intervals, OR 0.78 (95%CI 0.30–1.99). A post hoc sensitivity analysis of smoking abstinence at 6 months among those with complete data, controlling for patient nationality, gave similar results (OR 0.65 (95%CI 0.18–2.42)).

**Table 3:** Patient-Reported Smoking Abstinence, Quit Attempts, and Use of a Smoking Cessation Aid at 3-Weeks, 3-Months, and 6-Months Follow-Up, Adjusted for Clustering by PCP (*n*=34 Recruiting PCPs and *n*=287 Patients)

	Intervention (%)	Control (%)	Odds ratio (95%CI)*
Primary outcome			
7-day, point-prevalence smoking abstinence at 6-month follow-up, intention to treat sample	10/105 (9.5%)	19/182 (10.4%)	0.88 (0.37–2.10)
7-day, point-prevalence smoking abstinence at 6-month follow-up, complete case sample	10/85 (12%)	19/136 (14%)	0.78 (0.30–1.99)
Secondary outcomes			
7-day, point-prevalence smoking abstinence			
3-week follow-up	4/89 (4.5%)	11/156 (7%)	0.62 (0.18–2.17)
3-month follow-up	12/79 (15%)	16/134 (12%)	1.31 (0.57–3.02)
Quit attempt since last contact			
3-week follow-up	32/85 (38%)	33/144 (23%)	2.09 (1.04–4.20)
3-month follow-up	25/66 (38%)	34/118 (29%)	1.58 (0.76–3.26)
6-month follow-up	32/70 (46%)	40/115 (35%)	1.58 (0.86–2.90)
Use of a smoking cessation aid since last contact			
3-week follow-up	36/89 (40%)	36/156 (22%)	2.57 (1.21–5.45)
3-month follow-up	33/78 (42%)	36/134 (27%)	2.00 (1.11–3.60)
6-month follow-up	34/81 (42%)	40/135 (30%)	1.73 (0.94–3.18)

^*^Adjusted for clustering of patients at the level of general practitioners

There was no significant effect on smoking abstinence at any time point. The intervention increased the number of quit attempts at 3 weeks (OR 2.09, 95%CI 1.04–4.20) and the use of smoking cessation aids at the 3-week and 3-month follow-ups (OR 2.57, 95%CI 1.21–5.45 and OR 2.00, 95%CI 1.11–3.60, respectively). The CollaboRATE score, as reported 3 weeks after the baseline visit, was 8.05 (SD 2.25) with the intervention and 7.28 (SD 2.35) with the control PCPs (*p*=0.02).

## DISCUSSION

This pragmatic, cluster-randomized trial did not find a statistically significant impact of training PCPs to offer smoking cessation aids as the default choice using an encounter DA on patient-reported smoking abstinence at the 6-month follow-up. There were, however, significant increases in patient involvement in the baseline consultation, quit attempts at the 3-week follow-up, and use of smoking cessation aids at both 3-week and 3-month follow-ups. None of the outcomes was significant at the 6-month follow-up.

There are several possible explanations for our lack of impact on smoking abstinence. First is the lower than planned sample size, as most participating PCPs recruited fewer patients than anticipated. We sent e-mail reminders and tried to troubleshoot, but most PCPs found it difficult to find time to recruit eligible patients and did not feel comfortable delegating recruitment to their medical assistants. However, the 95% confidence interval around our primary outcome did not include our predicted odds ratio of 2.8 (abstinence 10.5% in the intervention arm versus 4% in the control arm). This suggests that even if we had recruited the planned sample size, we would not have demonstrated the anticipated effect. Another possibility is that our control intervention was stronger than anticipated. The landmark studies demonstrating the positive effect of training PCPs to offer smoking cessation advice generally had either control trainings on other topics, or no control training at all.^31^ PCPs in our control group received an in-person training about smoking cessation therapies and were not blinded to the nature of the data collection. It is also possible that the default choice approach increases the number of quit attempts with nicotine replacement therapy, as intended in our original logic model,^27^ but decreases the quality of quit attempts. We anticipated that a greater proportion of patients making quit attempts would be abstinent at 6 months because of the greater use of smoking cessation aids, which was not the case. It may be that more intensive interventions are needed, either with longer, repeated training interventions for PCPs, or improved follow-up of patients after the initial quit advice from the PCP. Our intervention was sufficient to improve intermediate outcomes compared to the control intervention, but this did not translate into increased cessation rate.

These results should be considered in the context of existing data about the default choice approach and DAs for smoking cessation. In a recent trial of nearly 1000 patients, intermediate outcomes improved but were not followed by increased cessation rate.^16^ There were strong increases in treatment use (60% vs 34%) and post-discharge counselling calls (89% vs 37%), but the 6% increase in verified smoking abstinence at 1 month (22% vs 16%) was not seen at 6 months (19% vs 18%).^16^ Other “opt-out” studies have been before-after studies.^15,32^ The current evidence does not clearly support use of the default choice approach. However, it is important to recall that the evidence to support motivational interviewing is also relatively weak,^33^ and it has been difficult to implement. Qualitative interviews, published separately, suggested that both PCPs and patients found our approach acceptable and useful in certain situations. We did not collect information on the time spent in consultation, and thus are unable to compare between groups. The best approach is likely to be the one that is feasible and sustainable in routine practice.

Strengths of this study included its pragmatic nature with an easily scalable, one-time training intervention and freely available encounter decision aid. Another strength was our choice of smoking abstinence as a primary outcome, rather than intermediary outcomes like decision quality or use of smoking cessation aids. We intervened in two countries with different contexts of primary care and reimbursement of medications, increasing the generalizability of our results. Limitations include PCP drop outs, PCPs who did not recruit the planned number of patients, and the relatively large imbalance in the number of patients recruited per PCP. However, the patient characteristics appeared to be balanced between arms, except for nationality, but a sensitivity analysis controlling for participant or PCP nationality did not change the results of the primary outcome. Varenicline was not at all, and bupropion only partially available during the study period, as Champix® (varenicline) was pulled from the market in 2021 and there were shortages of Zyban® (bupropion) during the study period. Furthermore, NRTs are not reimbursed by insurance in Switzerland, which could have decreased the appropriate use of these medications. Our primary outcome was self-reported smoking abstinence, without biochemical verification, which lowers its specificity; it is unclear whether this biased our results in one direction or towards a null result. Finally, due to the pragmatic nature of the trial, we did not specify how often PCPs should see their patients or measure the number and duration of visits during the 6 months between baseline and follow-up; this information could influence how to improve or implement our intervention.

In conclusion, an intervention training PCPs to offer smoking cessation aids as the default choice to current smokers in their consultation using an encounter DA did not increase self-reported smoking abstinence at 6 months follow-up. There were, however, promising improvements in shared decision-making and short-term increases in the number of quit attempts and use of nicotine replacement therapy. Given the limited efficacy and difficulties implementing current approaches to smoking cessation in primary care, these short-term increases and greater patient involvement in discussions should stimulate future research and partial implementation of our approach. For instance, a proactive discussion of treatment options using the decision aid could be an alternative approach to promoting quitting for some current smokers, while motivational interviewing remains the preferred approach for others. Future research could explore means of aiding patients who have initiated a quit attempt with their PCP.

## Supplementary Information

Below is the link to the electronic supplementary material.

Supplementary file1 (DOCX 294 KB)
