# Supplementary material for: Combining Default Choices and an Encounter Decision Aid to Improve Tobacco Cessation in Primary Care Patients: A Pragmatic, Cluster-Randomized Trial
Source: J Gen Intern Med. 2024 Oct 9;40(13):3078–85. doi: 10.1007/s11606-024-09088-9 (PMC12508413; doi:10.1007/s11606-024-09088-9)

**Combining Default Choices and an Encounter Decision Aid to Improve Tobacco Cessation in Primary Care Patients: A Pragmatic, Cluster-Randomized Trial**

Supplement

Supplementary Table 1: Baseline characteristics of general practitioners who recruited (n=34) or did not recruit patients (n=8)

| **GP Characteristics** | **Recruited (n=34)** | **Did not recruit (n=8)** | **Total (n=42)** |
| --- | --- | --- | --- |
| Age |  |  |  |
| 30-39 years | 11 (32%) | 3 (38%) | 14 (33%) |
| 40-49 years | 17 (50%) | 2 (25%) | 19 (45%) |
| 50-64 years | 6 (18%) | 3 (38%) | 9 (21%) |
| Gender |  |  |  |
| Men | 11 (32%) | 5 (63%) | 16 (38%) |
| Women | 23 (68%) | 3 (38%) | 26 (62%) |
| Practice country |  |  |  |
| Switzerland | 28 (82%) | 4 (50%) | 32 (76%) |
| France | 6 (18%) | 4 (50%) | 10 (24%) |
| Practice location |  |  |  |
| Urban | 25 (74%) | 6 (75%) | 31 (74%) |
| Rural | 9 (26%) | 2 (25%) | 11 (26%) |

Supplementary Figure 1: Number of patients recruited by individual general practitioners in the trial (n=42 GPs and 287 patients)


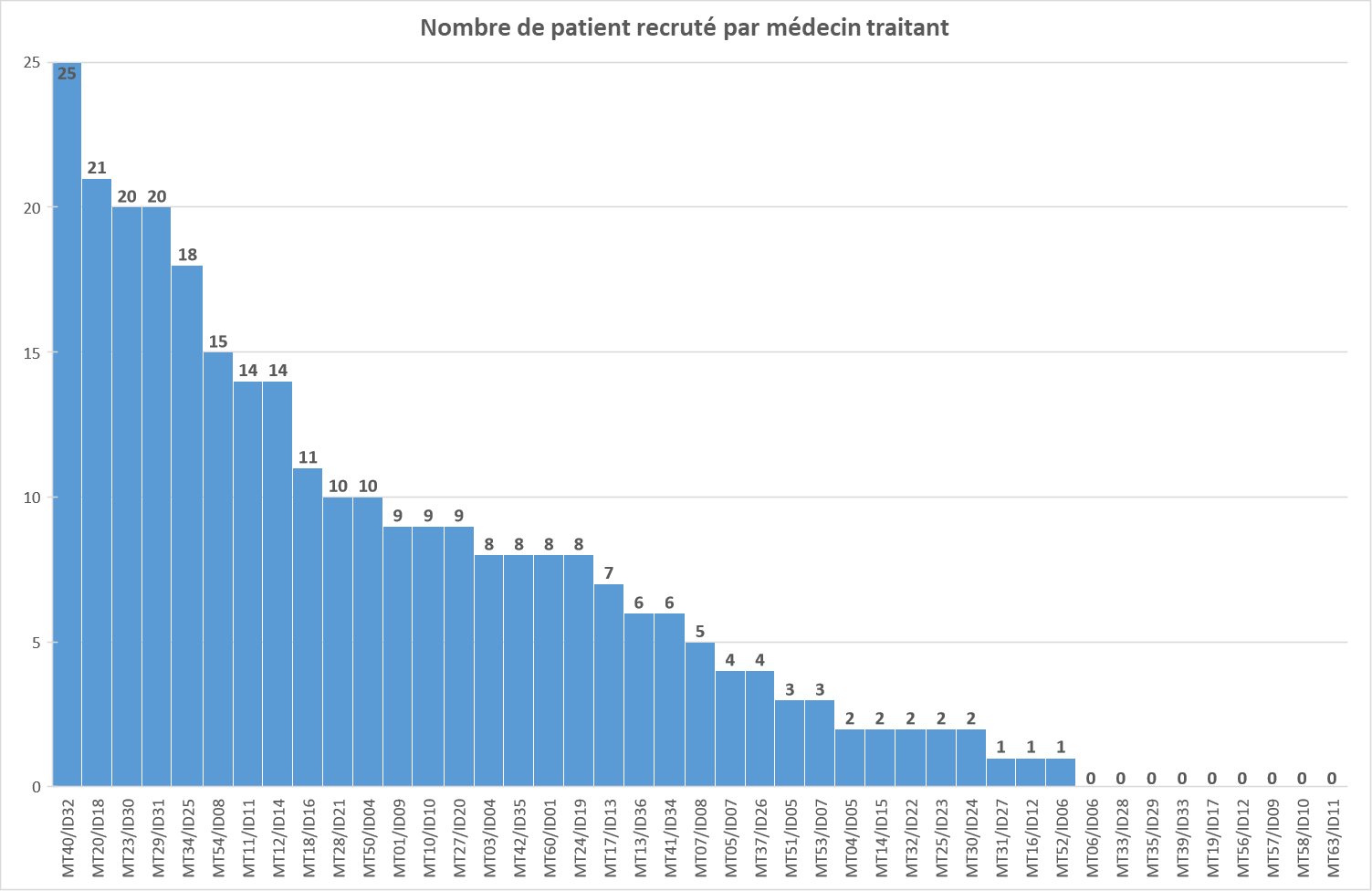


Supplemental Figure 2: The paper version of the decision aid translated into English. The original Decision aid is available here: <https://www.unisante.ch/fr/consultations-medicales/professionnels-sante/aides-decision>


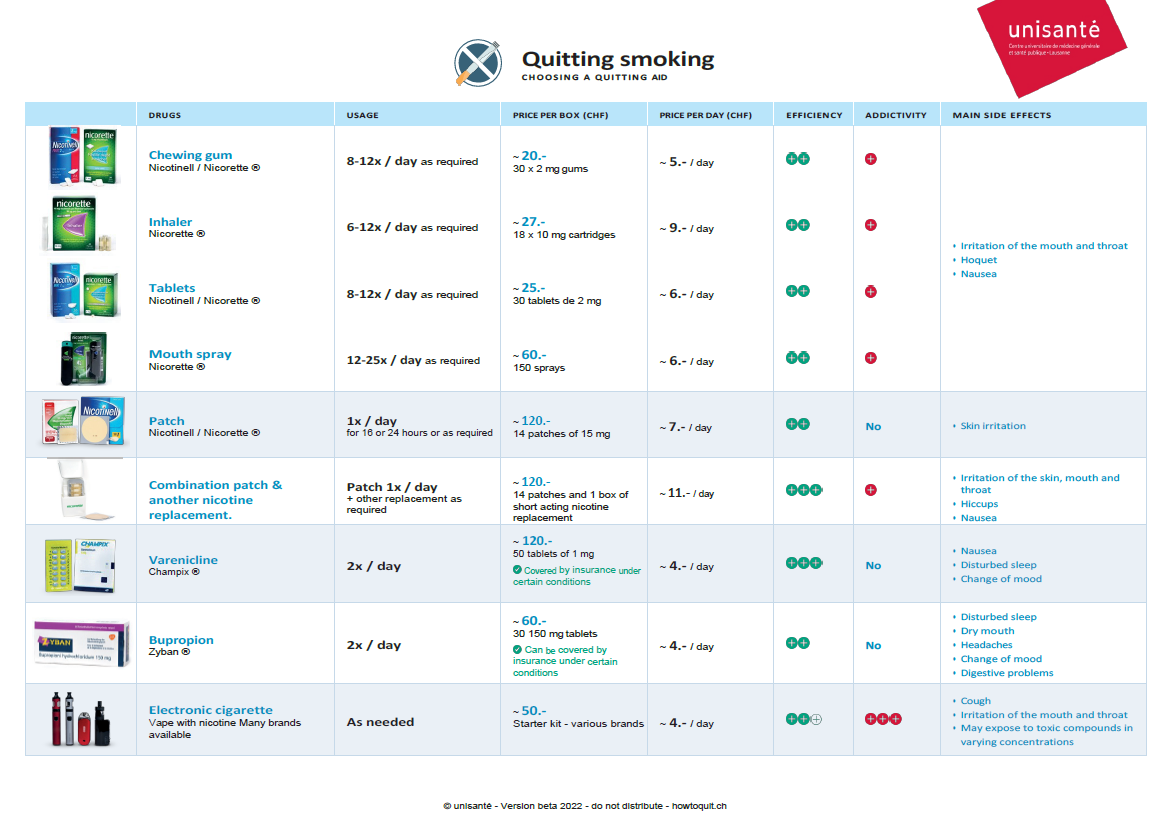

Supplement: Supplementary file 1 — Supplementary file1 (DOCX 294 KB) [file 11606_2024_9088_MOESM1_ESM.docx]
